# Breast Cancer Prognosis Risk Estimation Using Integrated Gene Expression and Clinical Data

**DOI:** 10.1155/2014/459203

**Published:** 2014-05-14

**Authors:** Ashish Saini, Jingyu Hou, Wanlei Zhou

**Affiliations:** School of Information Technology, Deakin University, 221 Burwood Highway, Melbourne, VIC 3125, Australia

## Abstract

*Background*. Novel prognostic markers are needed so newly diagnosed breast cancer patients do not undergo any unnecessary therapy. Various microarray gene expression datasets based studies have generated gene signatures to predict the prognosis outcomes, while ignoring the large amount of information contained in established clinical markers. Nevertheless, small sample sizes in individual microarray datasets remain a bottleneck in generating robust gene signatures that show limited predictive power. The aim of this study is to achieve high classification accuracy for the good prognosis group and then achieve high classification accuracy for the poor prognosis group. *Methods*. We propose a novel algorithm called the IPRE (integrated prognosis risk estimation) algorithm. We used integrated microarray datasets from multiple studies to increase the sample sizes (∼2,700 samples). The IPRE algorithm consists of a virtual chromosome for the extraction of the prognostic gene signature that has 79 genes, and a multivariate logistic regression model that incorporates clinical data along with expression data to generate the risk score formula that accurately categorizes breast cancer patients into two prognosis groups. *Results*. The evaluation on two testing datasets showed that the IPRE algorithm achieved high classification accuracies of 82% and 87%, which was far greater than any existing algorithms.

## 1. Introduction

The leading cause of death among breast cancer patients is from distant metastasis and recurrence [1]. The early stage prediction of prognosis after being diagnosed with breast cancer remains one of the significant challenges in breast cancer based clinical research. The accurate prediction of a good prognosis (low risk) or poor prognosis (high risk) of breast cancer patients based on metastasis or recurrence, can administer the decision for appropriate therapy. Patients categorized as low risk patients can be spared aggressive therapies, for example, chemotherapy, and can be treated with less toxic treatments, for example, tamoxifen or hormonal therapy. However, for patients categorized as high risk patients, chemotherapy or a combination of chemotherapy with other therapies may be the optimal therapy solution. These adjuvant therapies have high medical costs and also have vital short-term or long-term side effects [2]. Therefore, accurate classification of two prognosis groups can increase the survival rate of breast cancer patients and also reduce unnecessary costs associated with treatment. This can be achieved by developing robust prognostic tools that can help clinicians accurately categorize the prognosis group of patients and make effective and timely decisions about available treatments. Therefore, a challenge is to extract novel prognostic clinical or gene expression markers directly associated with breast cancer that can accurately categorize the two breast cancer prognosis groups.

During recent years, a wide number of disease markers have been identified from microarray-based gene expression profiles that have shown great potential in predicting the prognosis outcome for breast cancer [3–16]. Currently, treatment guidelines such as the US National Institutes of Health (NIH) [2] and St. Gallen [17] use clinical prognostic factors such as lymph node status, tumour size, and histologic grade in order to evaluate the distant metastasis risk for breast cancer patients. In breast cancer, Mammaprint or a 70-gene marker (or signature) [3] and a 76-gene signature [5], available commercially as a prognostic test [7], perform well in predicting metastasis that opposes the performance of clinical criteria. Even though these guidelines and the commercially available prognostic tests are popular, they are incapable of accurately characterizing breast cancer patients into high or low risk. As a result, many patients receive unnecessary adjuvant therapy, even when they can survive without it. Due to these issues, various groups now aim for an effective and robust gene expression signature that consists of genes with prognostic biological significance.

The results from existing studies raise some key questions. First, are the genes (from the gene signature) significantly associated with breast cancer or their metastasis? Second, if they are associated with breast cancer, are they cancer-causing genes (since a gene may be associated with cancer but not cause cancer)? Third, are the genes reproducible across different datasets or heavily depended on the chosen training set? Fourth, are the genes robust, providing similar classification results on different testing datasets? Fifth, do the genes represent the cancer related biological process GO terms or the pathways associated with the cancers?

The DNA microarray technology generating microarray gene expression datasets works as a robust tool in different aspects of cancer based research. The microarray dataset contains a low number of samples (~100–200) with a very large number of genes (~10,000–40,000) used as the training set. There is a greater chance of finding a gene signature with good predictive power on the training set and heavily degrades the performance on independent testing sets [9]. This is known as an over-fitted gene signature as the number of genes is far greater than the number of samples. Microarray based breast cancer gene expression datasets usually consist of a small number of samples due to the fact they are time consuming and expensive, which has always been an issue for accurately categorizing breast cancer prognosis groups [18]. Furthermore, at the time of predicting clinical outcomes, the sample size is reduced even further due to missing clinical factors for some of the samples in the microarray dataset [19]. This problem can be greatly reduced by integrating multiple microarray datasets from different studies that can increase the sample size, possibly in the order of thousands.

Shen et al. [20] and Teschendorff et al. [21] used “meta-analysis” to integrate different microarray datasets to develop breast cancer based prognostic gene signatures [9]. However, it is worthwhile noting that integrating microarray datasets from different studies is not so simple due to the different microarray platforms across different datasets, different experimental protocols, and different preprocessing methods. Therefore, in order to resolve all of these issues and be able to classify two prognosis groups of breast cancer patients effectively, it is essential to develop a novel breast cancer prognosis classifier that shows the good and stable classification performance from independent datasets.

In this study, we focus on utilizing an integrated dataset (that consists of a very large number of samples) along with clinical data to improve the performance of breast cancer prognosis classification. We define the two prognosis groups as good prognosis, which corresponds to the breast cancer-free state for at least 5 years, and the poor prognosis state, which corresponds to the recurrence of breast cancer or metastasis within 5 years. The prime goal of this study is to achieve high classification accuracy for the good prognosis group and then achieve high classification accuracy for the poor prognosis group. To accomplish this aim, we propose a breast cancer prognosis based classification algorithm by developing a virtual chromosome (VC) consisting of two components (correlation-factor and penalized-factor), which are used to extract our prognostic gene signature that consists of 79 genes. The extracted gene signature is then incorporated into a multivariate logistic regression model along with clinical variables for generating the risk score formula. A cut-off score of −1.480 was then used to classify the samples as a good-prognosis if the risk score was less than −1.480 and a poor-prognosis if the risk score was greater than or equals −1.480. We reported that when using the training dataset consisting of an equal number of two prognosis groups, it achieved a high classification performance in comparison to the training dataset that consisted of an unequal ratio of the two prognosis groups. The evaluation of our algorithm, called integrated prognosis risk estimation (IPRE), and the experimental comparisons with other prognosis based classifiers demonstrated that the IPRE algorithm outperforms other classifiers by achieving a high classification accuracy of 82% (with specificity of 88%) and 87% (with specificity of 95%) in the Desmedt [22] and the Vijver [3] datasets, respectively. This illustrates the effectiveness of the IPRE algorithm. In general, the IPRE algorithm achieves the following properties.

- High performance: achieves high classification performance in terms of high accuracy (and high specificity) for classifying the two prognosis groups.
- Dataset independence: achieves good results in the independent testing datasets (i.e., the dataset that was not used in the training dataset). This signifies the independency of the IPRE algorithm for any testing datasets or for any microarray platforms.
- Better classification: able to outperform the accuracy of seven other popular representative prognosis based algorithms.
- Biological significance: biological meaning of the IPRE algorithm based prognostic gene signature is significant and is related to the cancers.

## 2. Materials 

Seventeen breast cancer microarray gene expression datasets were downloaded from the publicly available NCBI GEO database [12] or supporting website [13] that has multiple platform types, that is, HG-U133A, HG-U133B, HG-U133PLUS 2.0, and the Agilent Human Genome. Overlapping samples or samples that have no clinical follow-up were removed from the datasets. The remaining samples were assigned either a high risk for metastasis/recurrence (if the metastasis/recurrence was observed within 5 years of follow-up) or low risk for metastasis/recurrence (if the metastasis/recurrence was not observed within 5 years of follow-up). The sample selection was independent of age, tumour grade, or any other clinical parameters. We have used normalized microarray datasets (log⁡_2_ intensity for single-channel platforms or log⁡_2_ ratio in dual channel platforms) as published by the original studies.

### 2.1. Training and Testing Datasets

Thirteen microarray gene expression datasets (see Table 1) were used as a training dataset to extract the prognostic gene signature, and the two microarray gene expression datasets, Desmedt dataset (GSE7390) [22] and the Vijver dataset [3], were used as a testing dataset for evaluating the performance of the algorithm. These datasets were selected first on the basis of metastasis or recurrence rate availability and then on tumour size availability. For patient samples with a missing estrogen receptor (ER) status and progesterone receptor (PR) status, the dichotomized ER gene (ESR1) and PR gene (PgR) mRNA (positive and negative) value was used instead. The probe expressions of the datasets were then converted to gene expressions as indicated by [23]. When multiple probes mapped the same gene, the mean of the probes was taken from a particular dataset, and the probes that began with “AFFX” were deleted, as there were no associated genes for these probes. The training and testing datasets were processed separately to ensure the independency of the testing datasets. The detailed information of the training and testing datasets is shown in Table 1.

### 2.2. Integrated Training Dataset

In this study, the task is to build a computational model that can accurately predict breast cancer patients with good prognosis or poor prognosis. As indicated by Sun et al. [24], larger-scale computational studies with more patient samples are required to best perform breast cancer prognosis given all available information. To achieve this goal, we integrate various training datasets by performing the following steps. First, the samples with missing gene expressions were deleted to remove any bias associated with them. Next, for any dataset *d*, the gene expression values were normalized between 0 and 1, by using (1) [24]

(1) $$\begin{array}{c} {\hat{e}}_{i}^{\left(g\right)}=\frac{e_{i}^{\left(g\right)}-e^{\min(g)}}{e^{\max(g)}-e^{\min(g)}}, \end{array}$$

where *e*_*i*_^(*g*)^ expresses the *g*th feature gene expression value for the *i*th sample and *e* ^min⁡⁡(*g*)^ and *e* ^max⁡⁡(*g*)^ represent the *g*th feature minimum and maximum gene expression value. This normalization mapped gene expression value is generated from different protocols into a uniform framework so the impact of the different protocols on the data integration can be reduced. Compared with the original data, the normalized gene expressions did not show any significant differences among study objects.

Further, a common list of genes from the distinct microarray platforms was extracted by cross-referencing each probe annotation in the microarray dataset. The cross-referencing of microarray gene expression data was done by the UniGene database [25]. To increase the dataset size (i.e., to overcome the “curse of dimensionality issue”), the list of common genes (i.e., 11,837 genes) from thirteen training microarray gene expression datasets was then directly integrated to have three unique Affymetrix platforms. This enables the gene signature to be independent of microarray datasets or their platform types. The integrated training dataset consists of GSE6532, GSE3494, GSE2990, GSE4922, GSE 31519, GSE 19615, GSE 1456, GSE 2603, GSE 2034, GSE 5327, GSE 12276, GSE 11121, and E-TABM-158. This makes a total of 13 datasets with 2,692 samples.

## 3. Integrated Prognosis Risk Estimation Algorithm 

The integrated dataset is used to construct the virtual chromosome (VC) in order to extract the prognostic gene signature. Furthermore, the clinical variables and our prognostic gene signature are incorporated in a multivariate logistic regression model which formed the risk score formula for predicting the prognosis outcome of breast cancer patients. Details of our IPRE algorithm are presented in the following sections.

### 3.1. Virtual Chromosome (VC)

We introduced a term called virtual chromosome (VC) which evaluates the prognostic score (observes significant differentially expressed gene expressions between two prognosis groups) of each gene in order to extract the prognosis based gene signature. A virtual chromosome consists of two components, that is, correlation factor (*α*) and penalized factor (*β*) (as shown in (5)). The prime concept of VC is to transform these two components into a combined value that reflects the prognostic significance of the genes.

In particular, *α* assesses the prognostic significance of each gene across all the samples in the integrated dataset. From the integrated dataset, we formed two groups. Group 1 had a metastasis or recurrence rate less than or equal to 5 years (most common point used in the literature) and Group 2 had a metastasis or recurrence rate greater than 5 years. The gene expression value of 0.5 (the middle value) was chosen as the probability measure, where the gene expressions less than 0.5 and greater than (or equal to) 0.5 were regarded as underexpressed and overexpressed gene expressions, respectively. To evaluate *α* for any gene, we first define the correlation-coefficient (cc) between metastasis or recurrence rate and gene expression as follows:

(2) cc(TTPi,eig) ={1;if  (TTPi≤5,  eig<0.5)OR  (TTPi>5,  eig≥0.5)−1;Otherwise,

where TTP_*i*_ defines the time to progression (i.e., metastasis or recurrence rate (in years)) for *i*th sample, *e*_*i*_^*g*^ defines gene expression of gene *g* in the *i*th sample, and cc(TTP_*i*_, *e*_*i*_^*g*^) defines the correlation-coefficient between TTP_*i*_ and *e*_*i*_^*g*^. Therefore, the correlation-factor (*α*) for any gene *g* can then be evaluated as

(3) $$\begin{array}{c} \alpha^{\left(g\right)}=\frac{1}{N}\left(p-q\right), \end{array}$$

where *N* defines the total number of samples in a dataset, *p* defines the total number of samples from the two groups that have the correlation-coefficient equaling 1, and *q* defines the total number of samples from the two groups that have the correlation-coefficient equaling −1. Here, *α* evaluates the difference between two prognostic groups from a microlevel, and the higher the *α* of a gene, the higher chance of being prognostically associated with breast cancer, and vice versa. However, *α* considered the microlevel dichotomized gene expression with dichotomized TTP (i.e., with two prognostic groups). Therefore, in order to reflect the actual difference between two prognostic groups, we used *β* (see (4)) that incorporated an actual gene expression level from a macro point of view.

Specifically, the penalized factor (*β*) penalizes each gene across all samples depending on the size difference between the two groups (i.e., Group 1 and Group 2, as mentioned above). For any gene *g*, we first evaluated the mean of each group based on the actual gene expression level, and the higher the difference between the mean of the two groups was, the lesser it was penalized, and vice versa. Mathematically, *β* for any gene *g* can then be evaluated as

(4) $$\begin{array}{c} \beta^{\left(g\right)}=1-\left|\frac{\left(\sum_{i=1}^{G_{1}}e_{i}^{\left(g\right)}\right)}{G_{1}}-\frac{\left(\sum_{j=1}^{G_{2}}e_{j}^{\left(g\right)}\right)}{G_{2}}\right|, \end{array}$$

where *G*_1_ and *G*_2_ define the total number of samples that belong to the Group 1 and Group 2, respectively, and *e*_*i*_^(*g*)^ and *e*_*j*_^(*g*)^ define the gene expression of gene *g* in the *i*th and *j*th sample, respectively. Here, *β* evaluates the actual difference between two prognostic groups from a macrolevel, and the higher the *β* of a gene is, the less chance the gene will be selected in our prognostic gene signature, and vice versa.

Therefore, to assess the overall prognostic significance of any gene *g* in the integrated dataset, their VC can be evaluated as the weighted combination of *α* and *β*; that is,

(5) $$\begin{array}{c} {\text{VC}}^{\left(g\right)}=\left({\tau_{1}*\alpha }^{\left(g\right)}\right)+\left(\tau_{2}*\beta^{\left(g\right)}\right), \end{array}$$

where *α* ^(*g*)^ and *β* ^(*g*)^ represent correlation factor and penalized factor for gene *g*, respectively, and *τ*_1_ and *τ*_2_ represent the weight associated with *α* and *β*, respectively. For the current setting, we assigned *τ*_1_ equals 1 and *τ*_2_ equals −1.0011, and these were chosen from our experimental analysis (i.e., by performing multivariate logistic regression analysis (see Section 3.3 for details)). The higher the VC score of a gene, the more chance of being selected in our prognostic gene signature, and vice versa. Finally, to extract the set of robust genes based on their VC score, we constructed the robust virtual chromosome.

### 3.2. Robust Virtual Chromosome

Our aim was to identify a set of robust genes with high VC scores in order to form our prognostic gene signature. For this purpose, we performed the following operations.

(i) First, we sorted the VC in descending order and then assigned a rank based on their VC values. The top gene shows the highest rank based on the highest VC value and the bottom gene shows the lowest rank based on the lowest VC value; that is,
  (6)  Sorted VC={VCn}; 1≤n≤φ s.t  VC1≥VC2≥VC3≥⋯≥VCφ,
  where VC_*n*_ defines the VC value for any gene that has been given *n*th rank and *φ* defines the total number of genes in the integrated dataset. In the sorted VC list, the top genes through to the bottom genes reflect the highly associated to the least associated differentially expressed genes in regards to breast cancer prognosis.
(ii) Next, while moving down the sorted VC list, we defined the robustness score (*R*) in order to identify how far the genes can be selected to form our prognostic gene signature. In other words, the robustness score for any set of genes defines the weighted sum of the genes VC with their weight which is evaluated as an inverse square root of the number of genes [26]. Specifically, the robustness score for any *m*th rank gene (*g*_*m*_) is
  (7) $$\begin{array}{c} R^{\left(g_{m}\right)}=R_{m}=\frac{1}{\sqrt{m}}\left(\overset{m}{\underset{n=1}{\sum }}{\text{VC}}_{n}\right). \end{array}$$
  The robustness score in the VC list first increases as it moves down, and then at a certain point achieves a maximum robustness score and thereafter starts decreasing.  Figure 2 illustrates the robustness score mechanism. The prime reason to generate a robustness score for the sorted VC list is to identify a set of top ranked robust genes that maximise the robustness score. Therefore, by using (7), the robustness score for each gene in the sorted VC list can be evaluated.
(iii) Finally, to extract the robust genes, we identified the robustness score that is the maximum in the sorted VC list. Therefore, the top gene until the gene whose robustness score is maximum formed our prognostic gene signature; that is,
  (8) Rmax⁡=max⁡1≤m≤φ⁡Rm,Our  Prognostic  Gene  Signature={gn}s.t R(gn)=Rn; R1≤Rn≤Rmax⁡.

### 3.3. Risk Score Estimation Using the Logistic Regression Model

The multivariate logistic regression model was constructed (that used clinical data and microarray data) in an attempt to robustly discover the predictors of prognosis risk for breast cancer patients.

For each sample in the integrated dataset, we first generated the gene expression mean of our prognostic gene signature and referred to this as the mScore. Next, for patient samples with a missing ER or PR status, the dichotomized ER gene (ESR1) and PR gene (PgR) mRNA (positive and negative) values were considered. We used the following influential variables in our multivariate logistic regression model, as mentioned in Table 2.

By using the variables shown in Table 2, the multivariate logistic regression model was constructed using the backward selection approach to form the risk score (RS) formula. The standard way to represent multivariate logistic regression equation is

(9) $$\begin{array}{c} {\text{Logit}\left(P\right)=\beta }_{0}+\beta_{1}X_{1}+\beta_{2}X_{2}+\cdots +\beta_{n}X_{n}, \end{array}$$

where Logit(*P*) = ln⁡⁡[*P*/(1 − *P*)], *P* is the estimated probability of breast cancer prognosis, *β*_0_ defines the constant, and *β*_*n*_ defines the regression coefficient for the *n*th variable (i.e., *X*_*n*_ variable). Here, *X*_*n*_ is a promoting factor if *β*_*n*_ > 0, and *X*_*n*_ is a suppressing factor if *β*_*n*_ < 0. A model with *X*_*n*_ that has a *P* value less than or equal to 0.05 was considered to be statistically significant.

The receiver operating characteristic (ROC) curves were used to assess the performance of the prediction model and determined an optimal cut-off point of *ϵ* that gives maximum sensitivity and specificity. Therefore, a sample can be classified as a good/poor prognosis if their RS is lesser/greater than the chosen cut-off point of *ϵ* from (9). The full algorithm workflow is shown in Figure 3.

## 4. Results and Discussion 

We conducted experiments on the integrated dataset of 2,692 samples (see Section 2.2). The samples with repetitions or a missing metastasis or recurrence rate were excluded. 2,268 samples remained, consisting of 897 poor prognosis breast cancer patients (metastasis or recurrence rate within five years) and 1,371 good prognosis breast cancer patients (metastasis or recurrence rate with more than five years). We named this integrated dataset the 2,268 dataset for illustrative purposes during our experiment.

Since the 2,268 dataset contained unequal numbers of samples in two prognosis groups, this may have caused algorithm bias towards the group with the higher number of samples. Therefore, to reduce the bias in the 2,268 dataset, we balanced both good and poor prognosis patient samples, that is, 897 poor prognosis breast cancer patients and 897 good prognosis breast cancer patients, with a total of 1,794 samples. We called this integrated dataset the 1,794 dataset for illustrative purposes during our experiment.

By applying our IPRE algorithm on the 1,794 dataset, we identified 79 breast cancer prognostic genes (log-rank test, value < 2*e* − 16) that formed our prognostic gene signature (see supplementary Table S1 in Supplementary Material available online at http://dx.doi.org/10.1155/2014/459203). The mScore (mean score of the gene signature) was then generated for each sample in the 1,794 dataset (see Section 3.3).

The “survival” package of the R-project (R Development Core Team, 2008) has been used to generate multivariate logistic regression analysis on the 1,794 dataset with variables such as ER, PR, HER2, tumour size, tumour grade, and mScore. As discussed earlier, we considered and selected variables that had a *P* ≤ 0.05 from our multivariate model and further used the risk score estimation to classify a sample as either a good or poor prognosis based on the score.  Table 3 lists the results of the variables (as mentioned in Table 2) from our multivariate analysis.

From Table 3, the tumour grade and the HER2 status variable were not selected as their *P* > 0.05. Therefore, tumour size, ER, PR, and the mScore were selected (being statistically significant) in our multivariate logistic regression model, and the risk score (RS) formula generated from our model used for predicting the prognosis of breast cancer patients was represented as

(10) RS=−0.99475−0.34534X2−0.19854X3 −0.21123X4−1.13115X6.

With (10), the risk score can be estimated for any sample that can classify breast cancer patients into either a good or poor prognosis using the chosen cut-off point of *ϵ*. From our experiments (see Section 4.1), we identified *ϵ* equals −1.480, which determines a sample to be a poor prognosis if their RS is greater than or equal to −1.480. However, a sample can be classified as a good prognosis if their RS is less than −1.480; that is,

 If RS ≥ −1.480,
   then it is a* poor prognosis *(*or high risk patients to breast cancer metastasis/recurrence*).
 Else, If RS < −1.480,
   then it is a* good prognosis *(*or low risk patients to breast cancer metastasis/recurrence*).

The classification results on the 1,794 dataset, the 2,268 dataset, and the two testing datasets, in addition to a comparison with other popular existing prognosis based algorithms, are detailed in the following subsections.

### 4.1. Classification Results on Integrated Training Datasets

We first performed experiments on an integrated training dataset, that is, 2,268 dataset, and then on the 1,794 dataset. The experiments were initially performed on a constructed risk score formula (10) with different cut-off points in order to choose the robust cut-off point that can characterize the two prognosis breast cancer patient groups with high accuracy, sensitivity, and specificity. We define sensitivity as the ability of the algorithm to identify the poor prognosis patients as poor-prognosis, specificity as the ability of the algorithm to identify the good prognosis patients as good-prognosis, and accuracy as the ability of the algorithm to accurately identify the patients as either good or poor prognosis patients. Our aim was not just to achieve high accuracy while ignoring sensitivity or specificity, but to achieve high sensitivity, specificity, and accuracy. It should be noted that we are more interested in achieving high specificity in order to spare the good prognosis patients from aggressive treatments. At the same time, we are interested in achieving high sensitivity in order to guide poor prognosis patients about the advanced treatment options available to them.

The supplementary Table S2 shows the classification results of our IPRE algorithm with different cut-off points in the 1,794 dataset and 2,268 dataset, respectively. The supplementary Table S2 shows that a cut-off point of −1.480 (in both the datasets) achieves high* F*-value, sensitivity, specificity, and accuracy, amongst others, and therefore −1.480 is chosen as a cut-off point in our risk score formula. Specifically, on the 1,794 dataset, the IPRE algorithm achieves 73%* F*-value, 77% accuracy, 63% sensitivity, and 91% specificity. On the other hand, for the 2,268 dataset, the IPRE algorithm achieves 59%* F*-value, 66% accuracy, 60% sensitivity, and 71% specificity. These results are reported in supplementary Table S2 (highlighted as bold) with their boxplots shown in supplementary Figure S1. Clearly, the 1,794 dataset that has equal proportions of the two prognosis groups shows much better results than the 2,268 dataset.

The overall performance of the model was assessed by drawing receiver operating characteristic (ROC) curve at different thresholds and by evaluating the area under the ROC curve (AUC). The larger the area under the ROC curve of an algorithm, the more effective the algorithm is. A model with an AUC of 0.5 is similar to flipping a coin. The ROC curve of an IPRE algorithm in the 1,794 dataset and the 2,268 dataset is shown in Figure 4.

The performance of the IPRE algorithm is good in the 1,794 dataset with an AUC of 0.770, whereas the algorithm performs average on the 2,268 dataset with an AUC of 0.688. This demonstrates that the 1,794 dataset was less biased than the 2,268 dataset, and therefore, was used to extract our prognostic gene signature and risk score formula (10) to classify the good and poor prognosis patient groups in the testing datasets.

Further, we compared our IPRE algorithm (that consists of clinical variables and gene signature) with IPRE (G) (that consists of gene signature) and IPRE (C) (that consists of clinical variables) on the 1,794 dataset and 2,268 dataset, respectively. The operations were similar to IPRE for the training and the construction of the risk-score formula for IPRE (G) and the IPRE (C), respectively.  Figure 5 shows the classification results of the IPRE, IPRE (G), and IPRE (C) algorithm, which clearly show the IPRE algorithm that consists of both genetic and clinical components outperforms IPRE (G), that consists of a genetic component only, and IPRE (C), that consists of a clinical component only. Also, IPRE (G) shows better results than IPRE (C), which shows the effectiveness of our gene signature.

### 4.2. Classification Results on Testing Datasets

To assess the performance of our model with a chosen cut-off point of −1.480 (see (10)) constructed with the IPRE algorithm, two testing datasets were used, that is, (a) the Desmedt dataset (GSE7390) [22] and (b) the Vijver dataset [3]. Comparisons with seven other existing prognosis based algorithms along with IPRE (G) and IPRE (C) were also performed to demonstrate that the IPRE algorithm was more effective and outperformed other existing algorithms. The algorithms are the 70-gene signature [3], the 21-gene signature [4], the 76-gene signature [5], the genomic grade index (GGI) [6], the invasiveness gene signature (IGS) [8], the 112-gene signature [9], and the interactome-transcriptome integration (ITI) [16].  Table 4 and Figure 6 show the classification results of the IPRE algorithm along with IPRE (G), IPRE (C), and seven other existing algorithms in the testing dataset of the Desmedt and the Vijver, respectively. Furthermore, the IPRE algorithm boxplots for the Desmedt and the Vijver dataset are shown in Figure 8. This clearly illustrates the separation between the two prognosis groups.

Table 4 and Figure 6 show that the IPRE and IPRE (G) algorithms achieved superior results compared to IPRE (C) and other existing popular prognosis based algorithms. Specifically, on the Desmedt dataset, the IPRE and IPRE (G) algorithms were able to achieve a prognosis classification accuracy of 82% (specificity of 88%) and 77% (specificity of 84%), respectively, which was significantly higher than the classification accuracies of IPRE (C), that is, 60% (specificity of 68%), and other existing algorithms, that is, 60% (specificity of 57%) for GGI, 40% (specificity of 28%) for 70 g, 54% (specificity of 53%) for 76 g, 52% (specificity of 42%) for 112 g, 58% (specificity of 59%) for IGS, 33% (specificity of 16%) for 21 g, and 72% (specificity of 82%) for ITI. Similar results were achieved from the Vijver dataset which shows the IPRE and IPRE (G) algorithms were able to achieve a prognosis classification accuracy of 87% (specificity of 95%) and 80% (specificity of 89%), respectively. This was much higher than the classification accuracies of IPRE (C) and other existing algorithms (see Table 4). From the two testing datasets, the IPRE algorithm achieved the highest accuracy and performed better than any of the others, including IPRE (G), IPRE (C), and other existing algorithms. It should be noted that if comparing existing algorithms in the Desmedt dataset, ITI showed the second best results (in terms of accuracy) and GGI ranked third. On the other hand, in the Vijver dataset, GGI ranked second and ITI ranked fifth. This points to the fact that the classification accuracy of existing algorithms heavily depends on their choice of training dataset. Therefore, it can be true to say that existing algorithms do not show consistent performances across distinct microarray datasets as these algorithms are biased towards the datasets used for generating gene signatures. However, by increasing the training compendia to a large number of samples with multiple microarray platforms, the algorithm's dependency on the datasets or microarray platforms can be greatly reduced. This was demonstrated by the IPRE algorithm as we incorporated multiple training datasets with a very large number of samples across multiple microarray platforms. Therefore, these results concluded that the IPRE algorithm was stable and robust against any testing dataset or their microarray platform in order to categorise good prognosis (low risk) versus poor prognosis (high risk) breast cancer patients.

Further, to fairly evaluate and compare the performance of existing algorithms by removing any differences that come with clinical variables or their gene signatures, the logistic regression model was constructed (that used clinical variables and a gene signature) for each existing algorithm in a similar way as constructed for IPRE (see Section 3.3). For simplicity, we still denote the existing algorithms as IGS, 21 g, 112 g, 70 g, 76 g, GGI, and ITI.  Figure 7 shows the classification performance of IPRE and other existing algorithms constructed using the logistic regression model with considered clinical variables and their respective gene signature, and also shows that the IPRE algorithm achieved higher specificity and accuracy that outperformed other existing algorithms. For each of the existing algorithms, these experiments concluded that integrating clinical variables and gene signatures (see Figure 7) showed better classification performances than considering only gene signatures (see Figure 6). Furthermore, these experiments conducted using different angles showed the effectiveness of the proposed algorithm in classifying the two prognosis groups.

Venet et al. [35] compared 47 breast cancer gene signatures to the signatures made by random genes and claimed that most random gene expression signatures are significantly associated with the breast cancer outcome. Their study showed that almost 60% of existing gene signatures were not significantly better outcome predictors than the random gene signatures that contain 1,000 gene signatures of identical size, and nearly 23% of them were worse predictors. Therefore, to compare the IPRE algorithm against random gene signatures, we constructed 1,000 random gene signatures of identical size. For fair evaluation with the IPRE algorithm, we incorporated the clinical variables with the random gene signatures and called it RAND.

Figure 9 reveals that the IPRE algorithm showed a significantly stronger association with distant metastasis free survival (DMFS) outcome than the median of the random gene signatures (RAND) in the Desmedt dataset and the Vijver dataset, respectively. The classification results of the RAND and IPRE algorithms in the Desmedt dataset and the Vijver dataset, as shown in Table 5, clearly show that the IPRE algorithm significantly outperformed RAND.

**Figure 1:**
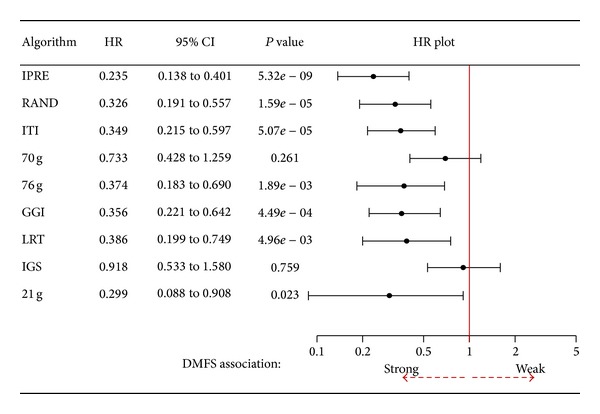
Hazard ratios for comparison of distant metastasis free survival (DMFS) association of IPRE and other algorithms in the Desmedt dataset. Here, HR stands for hazard ratio and 95% CI represents 95% confidence intervals. The lesser the HR is, the stronger the survival association of the algorithm is. Many existing published gene signatures and random gene signatures of identical size are not better outcome predictors than IPRE.

**Figure 2:**
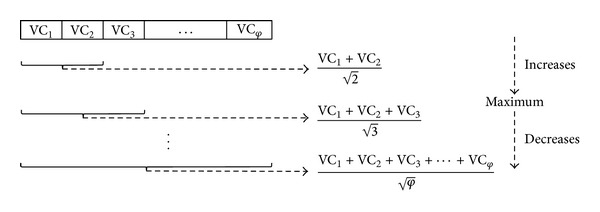
Robustness score overview. Here, VC_*φ*_ represents the VC for any *φ*th ranked gene. The robustness score in the list increases first until a certain point and then achieves a maximum robustness score, and thereafter, begins to decrease.

**Figure 3:**
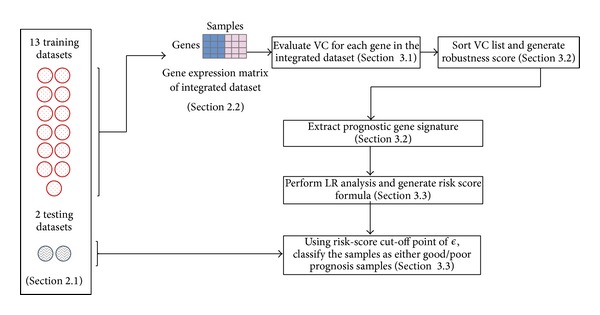
IPRE algorithm workflow.

**Figure 4:**
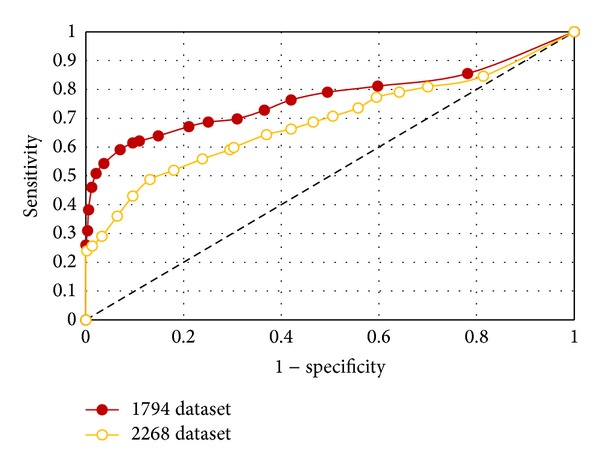
ROC curves of the IPRE algorithm in the 1,794 dataset and the 2,268 dataset. Clearly, this validates the 1,794 dataset as better compared to the 2,268 dataset.

**Figure 5:**
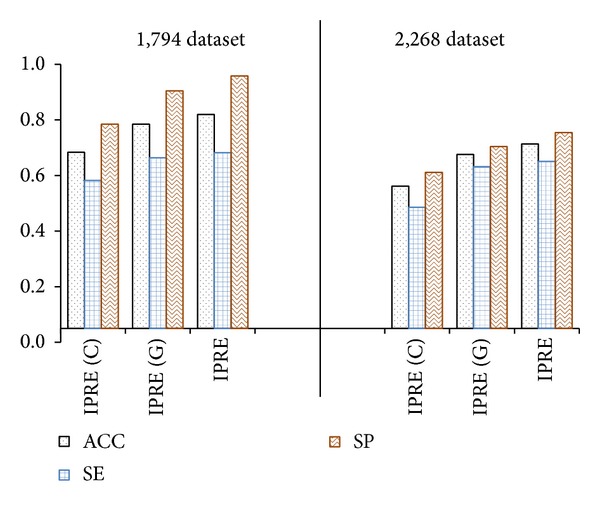
Classification results of the IPRE (C), IPRE (G), and IPRE algorithm on the 1,794 and 2,268 dataset. Here, ACC defines accuracy, SE defines sensitivity, and SP defines specificity.

**Figure 6:**
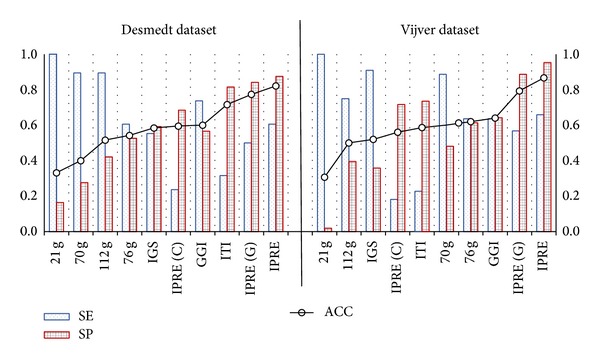
Classification results of IPRE, IPRE (G), IPRE (C), and seven other existing algorithms on the Desmedt and Vijver dataset. Here, SE defines sensitivity, SP defines specificity, and ACC defines accuracy.

**Figure 7:**
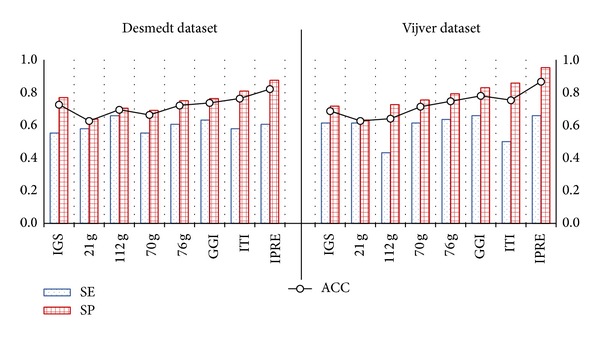
Classification results of IPRE and seven other existing algorithms constructed with a logistic regression model (with integrated clinical variables and their gene signature) on the Desmedt and Vijver dataset.

**Figure 8:**
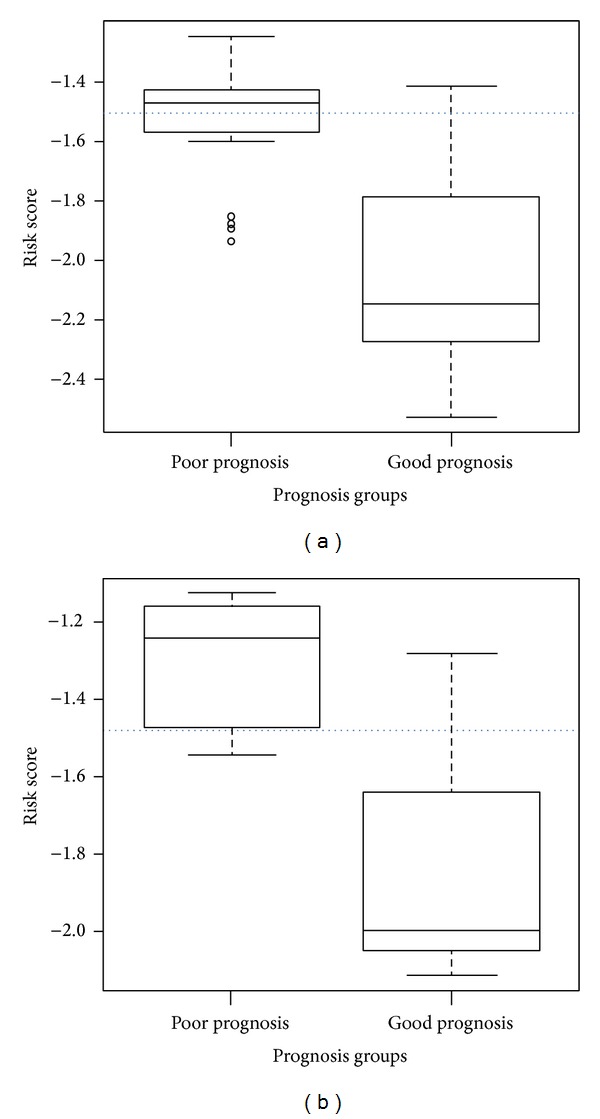
The boxplot of the IPRE algorithm for two prognosis groups, that is, poor-prognosis and good-prognosis. The left figure represents the boxplot for the Desmedt dataset and the right figure represents the boxplot for the Vijver dataset. Here, the *x*-axis represents the two prognosis groups, the *y*-axis represents the risk scores, and the dotted horizontal line represents our chosen cut-off point, that is, −1.480. It can be clearly seen that the two prognosis groups are separated in both datasets.

**Figure 9:**
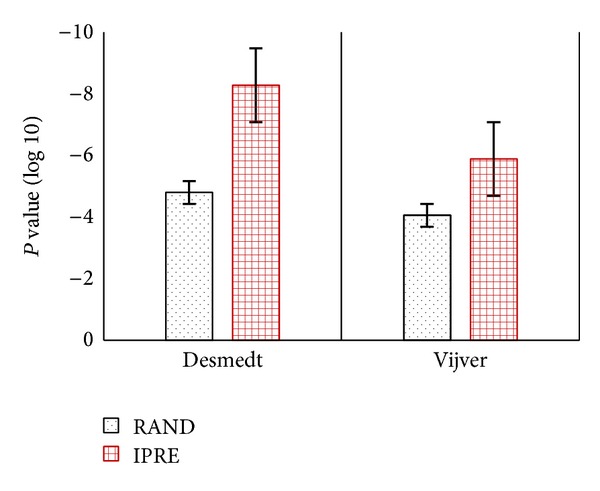
Bar-plot representing the *P* value of the distant metastasis free survival (DMFS) outcome for RAND and IPRE in the Desmedt and Vijver dataset, respectively. Here, the *P* value of RAND represents the median *P* value generated from the random gene signatures.

**Figure 10:**
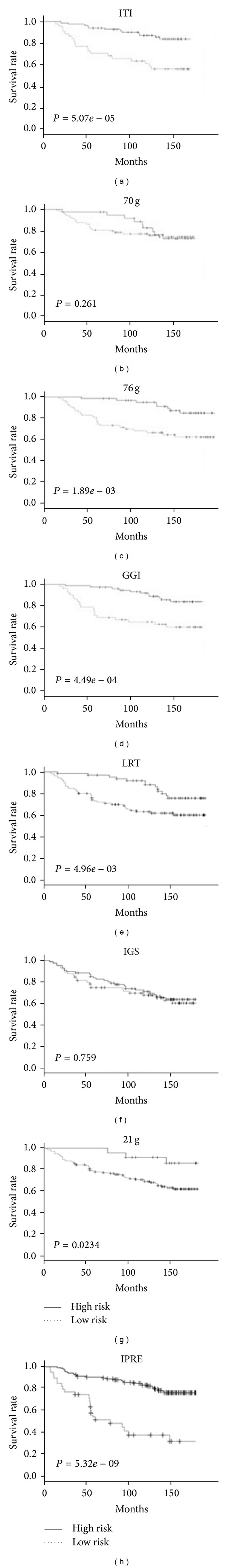
Kaplan-Meier survival graphs for good and poor prognosis groups in the Desmedt dataset for the IPRE algorithm and seven other existing algorithms. A log-rank test was performed to assess the *P* value and to signify that the lower the *P* value, the better the separation between the two prognosis groups. From this figure, the IPRE algorithm was able to achieve the lowest *P* value and perform the best at separating the two prognosis groups.

To get the overall picture of the performance comparison of IPRE and other existing algorithms, we calculated hazard ratios using all combinations of DMFS end-points and cohorts in the Desmedt dataset, as shown in Figure 1 with their related survival analysis discussed below (see Figure 10). Our analysis concludes that the IPRE algorithm was a significantly better outcome predictor than random gene signatures of an identical size. However, the random gene signatures showed significantly better outcomes than other published gene signatures used in our study. These results further validate the findings of Venet et al. [35] who suggest most existing gene signatures are not significantly better outcome predictors than the random gene signatures of identical size.

Next, a Kaplan-Meier survival analysis is performed on the Desmedt dataset by using the “survival” package of the R-project (R Development Core Team, 2008). Figure 10 shows the survival curves of the IPRE algorithm and seven other existing algorithms using the DMFS rate. The Log-rank statistical test gave a *P* value of 5.32*e* − 09 for the IPRE algorithm, which was statistically significant (i.e., value ≤ 0.05), and indicated the best separation between the two prognosis groups (i.e., good prognosis or low risk to distant metastasis, and poor prognosis or high risk to distant metastasis) as compared with other existing algorithms. It should be noted that the 70 g and IGS algorithms were incapable of separating patients into significant prognostic groups (*P*  value > 0.05). These results supported our previous conclusions that the IPRE algorithm was robust enough to separate patients into two significant prognosis groups.

### 4.3. Our Prognostic Gene Signature Stability with Existing Gene Signatures

We performed stability analysis for assessing the overlap between existing gene signatures and our prognostic gene signature.  Table 6 shows the number of overlapped genes from our prognostic gene signature compared with other existing signatures.

Our prognostic gene signature shows the overlap with existing gene signatures within the range of 1–21%. We discovered that gene signatures extracted from the multiple training datasets, such as ITI, performed better and showed greater overlap with our gene signature, that is, 21%. This overlap was significantly greater compared to other gene signatures generated from a lower number of training samples. This demonstrates the effectiveness of incorporating a larger number of patient samples in a training dataset with multiple microarray platforms to enhance the performance of the algorithm across a wide range of testing datasets.

### 4.4. Biological Analysis of Our Prognostic Gene Signature

The biological analysis of our prognostic gene signature was performed using the gene ontology (GO) analysis and pathway analysis. For each gene in our prognostic gene signature, GO analysis was performed using the enriched biological process GO terms [36]. The pathway analysis was performed using the KEGG pathways [37].

First, we input genes in our prognostic gene signature into the program called Database for Annotation, Visualization, and Integrated Discovery (DAVID) [33]. DAVID then produced the enriched biological process GO terms and the associated KEGG pathways with their *P* values computed using hypergeometric distribution. This program was helpful to identify the significant GO terms and KEGG pathways. The supplementary Tables S3 and S4 represent the enriched GO terms and the enriched KEGG pathways for our prognostic gene signature, respectively.

From supplementary Table S3, it can be seen that several enriched GO terms are associated with the processes that were seen to be disrupted in the cancers, such as regulation of apoptosis, regulation of transcription, cell proliferation, positive regulation of cell activation, and positive regulation of immune response, besides many others. Furthermore, the supplementary Table S4 shows that the pathways of our prognostic gene signature were related to the cancers, such as apoptosis, cell cycle, integrin signalling pathway, T cell activation, ATM signalling, and pathways in cancer. These results demonstrate that our prognostic gene signature consists of biologically significant genes that are biologically associated with the cancers. The supplementary Figure S2 further illustrates the biological meaning of our prognostic gene signature by showing the KEGG cell cycle pathway and the genes in our gene signature (as highlighted in red) that appear in this pathway. It was observed that many genes in our prognostic gene signature were associated with the KEGG cell cycle pathway.

We also observed that various genes in our prognostic gene signature were previously recognised as oncogenes, such as, CD74, GATA3, CD24, and C1S, whose altered expression patterns were associated with different prognosis states or with various types of cancers [38–41]. However, for other genes not previously reported as oncogenes, they may be innovative oncogenes that promote breast cancer or its aggressiveness. These facts conclude that our prognostic gene signature is biologically meaningful and relates to breast cancer prognosis.

## 5. Conclusion

We proposed a prognosis based classification algorithm to categorize breast cancer patients as high versus low risk. We developed a virtual chromosome to extract our prognostic gene signature that consists of 79 differentially expressed genes. The current study particularly focused on achieving high specificity, at the same time as achieving high sensitivity. For this, a multivariate logistic regression model was then used to correlate the microarray data (our prognostic gene signature) and clinical data (tumour size, ER, PR) to generate the risk score estimation formula. A −1.480 cut-off point was chosen to distinguish two prognosis groups of breast cancer patients.

We formed two integrated training datasets, that is, the 1,794 dataset and the 2,268 dataset, which consisted of multiple training datasets with a very large number of patient samples and multiple microarray platforms so the training size was large enough to reduce any bias associated with training datasets of a small size and to lessen the dependency of the algorithm on any particular training dataset. From our experiments, we concluded the integrated training dataset (i.e., 1,794 dataset) with equal number of good and poor prognosis patient samples which was less biased and achieved superior performance compared to the integrated training dataset (i.e., 2,268 dataset) with unequal numbers of good and poor prognosis patient samples.

Unlike other algorithms, we observed that the classification performance of our IPRE algorithm on multiple testing datasets was consistently good, suggesting that the IPRE algorithm was independent of any testing dataset when it achieved high classification performance. Using the two testing datasets (the Desmedt and Vijver dataset), we demonstrated that the IPRE algorithm achieved its highest classification performance by characterizing a high number of good and poor prognosis breast cancer patients, and it was able to outperform random gene signatures of identical size and seven other popular prognosis based classification algorithms (see Table 4). This concludes that the IPRE algorithm was robust and effective in classifying the two prognosis groups on any dataset without depending on any other factor. The IPRE algorithm based prognostic gene signature was also validated biologically using GO terms and pathway classes; this shows that the biological meaning of our prognostic gene signature was significant. These results concluded that the IPRE algorithm was significant in achieving high specificity (and also high sensitivity), which was the initial aim of our study so that low risk patients avoid unnecessary or aggressive therapies.

The above discussions and results suggest that the proposed algorithm has the potential to guide physicians at the early decision stage of breast cancer prognosis. Further, a phase-2 clinical trial would be advisable to categorize the two prognosis groups of breast cancer using the IPRE algorithm.

## Supplementary Material

The Supplementary Material consists of six files, including Table S1, S2, S3, and S4 and Figure S1 and S2. The Table S1 shows the proposed algorithm based prognostic gene signature. The Table S2 shows the prognosis based average classification results of IPRE algorithm with different cut-off points. The Table S3 and S4 shows the enriched gene ontology (GO) terms and the enriched pathways for the genes in the IPRE gene signature. At last, the Figure S1 shows the boxplot of the IPRE algorithm, and the Figure S2 shows the KEGG pathway of a cell cycle. These supplementary files further supports that the IPRE gene signature has its advantage in classifying two prognosis groups for breast cancer patients effectively.

## Figures and Tables

**Table 1:** Breast cancer microarray gene expression datasets used in this study.

Data set	Country	Poor/good prognosis	Samples	Platform	IHC	References
GSE1456*	Sweden	41/118	159	HG-U133A, HG-U133B	NA	[2]
GSE2990*	Sweden, UK	37/85	189	HG-U133A	ER	[7]
GSE6532*	Belgium	56/61, 100/150, 9/68	327, 255, 87	HG-U133A, HG-U133B, HG-U133Plus 2.0	ER, PR	[11]
E-TABM-158*	USA	54/63	118	HG-U133A	ER, PR, HER2	[13]
GSE5327*	USA	11/47	58	HG-U133A	ER	[17]
GSE2603*	USA	36/46	121	HG-U133A	ER, PR	[18]
GSE31519*	Germany	52/10	67	HG-U133A	ER, PR, HER2	[22]
GSE3494*	Sweden	60/176	251	HG-U133A, HG-U133B	ER, PR	[27]
GSE4922*	Singapore	91/158	255	HG-U133A, HG-U133B	ER	[28]
GSE19615*	USA	14/0	115	HG-U133Plus 2.0	ER, PR, HER2	[29]
GSE2034*	Netherlands	106/180	286	HG-U133A	ER	[30]
GSE12276*	Netherlands	190/14	204	HG-U133Plus 2.0	NA	[31]
GSE11121*	Germany	47/153	200	HG-U133A	NA	[32]
GSE7390^§^	Europe	44/154	198	HG-U133A	ER	[33]
van de Vijver^§^	Netherlands	84/211	295	Agilent Human Genome	ER	[34]

Total: 15	8 countries	1,042/1,694	3,185	4 platforms		

*Represents the training datasets and ^§^represents the testing datasets.

**Table 2:** Variables used in our multivariate logistic regression model.

Variable	Characteristics		Code
*X*_1_	Tumour grade	(I-II) (III)	0 1
*X*_2_	Tumour size (cm)	(<2) (2-3) (≥4)	0 0.55 1
*X*_3_	ER (estrogen receptor)	(−) (+)	−1 1
*X*_4_	PR (progesterone receptor)	(−) (+)	−1 1
*X*_5_	HER2 (human epidermal growth factor receptor 2)	(−) (+)	−1 1
*X*_6_	mScore (mean of our prognostic gene signature)	(0 to 1)	NA

**Table 3:** Multivariate analysis of the variables in the 1,794 dataset.

Variable	Characteristics	*β*	*z*	SE	*P*	Selected
*X*_1_	Grade	−0.06176	−0.674	0.09163	0.50	No
*X*_2_	Size	−0.34534	−3.029	0.11401	0.002	Yes
*X*_3_	ER	−0.19854	−4.441	0.04471	8.95*E* − 06	Yes
*X*_4_	PR	−0.21123	−4.356	0.04849	1.33*E* − 05	Yes
*X*_5_	HER2	0.08819	1.761	0.05007	0.08	No
*X*_6_	mScore	−1.13115	−12.918	0.08757	2.00*E* − 16	Yes
	Constant	−0.99475				

**Table 4:** Prognosis based classification results of the IPRE, IPRE (G), IPRE (C), and seven other existing algorithms in the testing dataset of (a) the Desmedt and (b) the Vijver.

Algorithm		*N*	TN	FP	TP	FN	SE (95% CI)	SP (95% CI)	ACC (95% CI)
(a) Desmedt dataset	GGI	190	86	66	28	10	0.737 (0.60 to 0.88)	0.566 (0.49 to 0.64)	0.600 (0.53 to 0.67)
	70 g	190	42	110	34	4	0.895 (0.80 to 0.99)	0.276 (0.21 to 0.35)	0.400 (0.33 to 0.47)
	76 g	190	80	72	23	15	0.605 (0.45 to 0.76)	0.526 (0.45 to 0.61)	0.542 (0.47 to 0.61)
	112 g	190	64	88	34	4	0.895 (0.80 to 0.99)	0.421 (0.34 to 0.50)	0.516 (0.45 to 0.59)
	IGS	190	90	62	21	17	0.553 (0.39 to 0.71)	0.592 (0.51 to 0.67)	0.584 (0.51 to 0.65)
	21 g	190	25	127	38	0	1 (1.00 to 1.00)	0.164 (0.11 to 0.22)	0.332 (0.27 to 0.40)
	ITI	190	124	28	12	26	0.316 (0.17 to 0.46)	0.816 (0.75 to 0.88)	0.716 (0.65 to 0.78)
	IPRE (C)	190	104	48	9	29	0.237 (0.10 to 0.37)	0.684 (0.61 to 0.76)	0.595 (0.53 to 0.67)
	IPRE (G)	190	128	24	19	19	0.500 (0.34 to 0.66)	0.842 (0.78 to 0.90)	0.774 (0.71 to 0.83)
	IPRE	**190**	**133**	**19**	**23**	**15**	**0.605** (**0.45 to 0.76**)	**0.875** (**0.82 to 0.93**)	**0.821** (**0.77 to 0.88**)

(b) Vijver dataset	GGI	150	68	38	28	16	0.636 (0.49 to 0.78)	0.642 (0.55 to 0.73)	0.640 (0.56 to 0.72)
	70 g	150	51	55	39	5	0.886 (0.79 to 0.98)	0.481 (0.39 to 0.58)	0.600 (0.52 to 0.68)
	76 g	150	65	41	28	16	0.636 (0.49 to 0.78)	0.613 (0.52 to 0.71)	0.620 (0.54 to 0.70)
	112 g	150	42	64	33	11	0.750 (0.62 to 0.88)	0.396 (0.30 to 0.49)	0.500 (0.42 to 0.58)
	IGS	150	38	68	40	4	0.909 (0.82 to 0.99)	0.358 (0.27 to 0.45)	0.520 (0.44 to 0.60)
	21 g	150	2	104	44	0	1 (1.00 to 1.00)	0.019 (−0.01 to 0.04)	0.307 (0.23 to 0.38)
	ITI	150	78	28	10	34	0.227 (0.10 to 0.35)	0.736 (0.65 to 0.82)	0.587 (0.51 to 0.67)
	IPRE (C)	150	76	30	8	36	0.182 (0.07 to 0.30)	0.717 (0.63 to 0.80)	0.560 (0.48 to 0.64)
	IPRE (G)	150	94	12	25	19	0.568 (0.42 to 0.71)	0.887 (0.83 to 0.95)	0.793 (0.73 to 0.86)
	IPRE	**150**	**101**	**5**	**29**	**15**	**0.659** (**0.52 to 0.80**)	**0.953** (**0.91 to 0.99**)	**0.867** (**0.81 to 0.92**)

Here, *N* defines the total number of samples, TP defines true positive, TN defines true negative, FP defines false positive, FN defines false negative, SE defines sensitivity, SP defines specificity, ACC defines accuracy, and 95% CI defines 95% confidence intervals. Due to space restrictions, we represent the genomic grade index as GGI, 70-gene signature as 70 g, 76-gene signature as 76 g, 112-gene signature as 112 g, invasiveness gene signature as IGS, 21-gene signature as 21 g, and interactome-transcriptome integration as ITI. From the table, the IPRE algorithm achieved superior performance amongst others (as highlighted in bold) in the Desmedt dataset and the Vijver dataset.

**Table 5:** Prognosis based classification results of the RAND and IPRE algorithms in the testing dataset of (a) the Desmedt and (b) the Vijver.

Algorithm		*N*	TN	FP	TP	FN	SE (95% CI)	SP (95% CI)	ACC (95% CI)
(a) Desmedt dataset	RAND	190	128	24	23	15	0.605 (0.45 to 0.76)	0.842 (0.78 to 0.90)	0.795 (0.74 to 0.85)
	IPRE	**190**	**133**	**19**	**23**	**15**	**0.605** (**0.45 to 0.76**)	**0.875** (**0.82 to 0.93**)	**0.821** (**0.77 to 0.88**)

(b) Vijver dataset	RAND	150	91	15	26	16	0.619 (0.47 to 0.77)	0.858 (0.79 to 0.92)	0.791 (0.73 to 0.86)
	IPRE	**150**	**101**	**5**	**29**	**15**	**0.659** (**0.52 to 0.80**)	**0.953** (**0.91 to 0.99**)	**0.867** (**0.81 to 0.92**)

**Table 6:** The number (percentage) of genes in our prognostic gene signature that matches existing gene signatures.

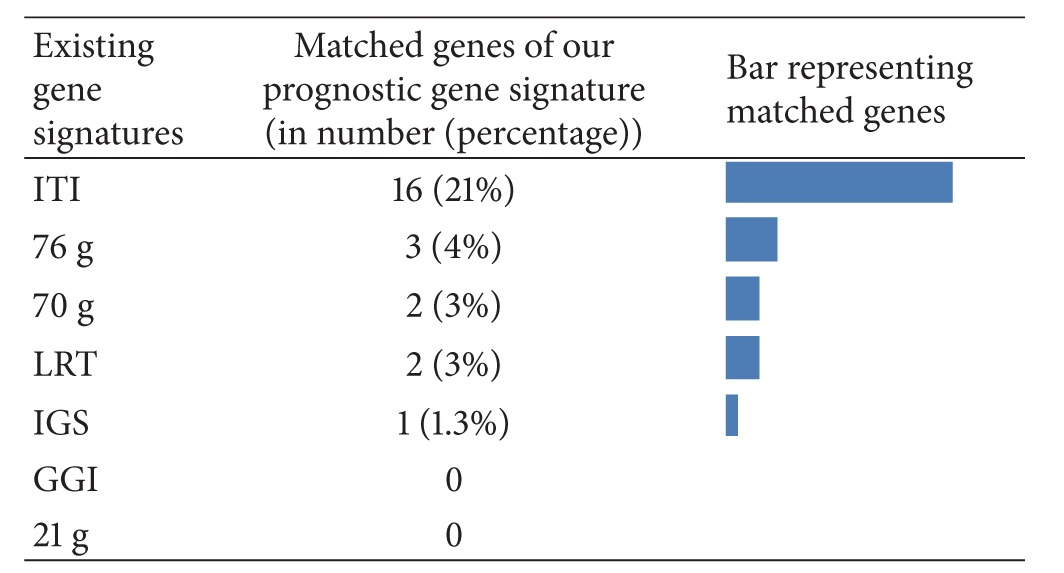

The last column represents the bar graph of our matched genes (in percentage) with other gene signatures. Here, the ITI gene signature shows the maximum overlap (21%) amongst other representative gene signatures.
