# Supplementary material for: Breast Cancer Prognosis Risk Estimation Using Integrated Gene Expression and Clinical Data
Source: Biomed Res Int. 2014 May 14;2014:459203. doi: 10.1155/2014/459203 (PMC4052785; doi:10.1155/2014/459203)
Supplement: Supplementary file 1 — The Supplementary Material consists of six files, including Table S1, S2, S3, and S4 and Figure S1 and S2. The Table S1 shows the proposed algorithm based prognostic gene signature. The Table S2 shows the prognosis based average classification results of IPRE algorithm with different cut-off points. The Table S3 and S4 shows the enriched gene ontology (GO) terms and the enriched pathways for the genes in the IPRE gene signature. At last, the Figure S1 shows the boxplot of the IPRE algorithm, and the Figure S2 shows the KEGG pathway of a cell cycle. These supplementary files further supports that the IPRE gene signature has its advantage in classifying two prognosis groups for breast cancer patients effectively. [file 459203.f1.pdf]

Supplementary Material

Supplementary Tables

Table S1. Our prognostic gene signature consists of 79 genes.

Gene Signature	Probe Name	Entrez Gene ID	Cytoband	Description	Correlation-factor (α)	Penalized-factor (β)
**KCTD12**	212188_at	115207	13q22.3	potassium channel tetramerisation domain containing 12	0.4002	0.7911
**LOC93349**	214791_at	93349	2q37.1	hypothetical protein BC004921	0.3880	0.7812
**C1S**	208747_s_at	716	12p13	complement component 1, s subcomponent	0.3846	0.7481
**SERPING1**	200986_at	710	11q12-q13.1	serpin peptidase inhibitor, clade G (C1 inhibitor), member 1, (angioedema, hereditary)	0.3824	0.7846
**BST2**	201641_at	684	19p13.2	bone marrow stromal cell antigen 2	0.3824	0.7726
**TNFSF10**	202687_s_at	8743	3q26	tumor necrosis factor (ligand) superfamily, member 10	0.3824	0.7898
**HLA-DPA1**	211990_at	3113	6p21.3	major histocompatibility complex, class II, DP alpha 1	0.3813	0.7989
**CXCL12**	203666_at	6387	10q11.1	chemokine (C-X-C motif) ligand 12 (stromal cell-derived factor 1)	0.3813	0.7707
**C3**	217767_at	718	19p13.3-p13.2	complement component 3	0.3757	0.7561
**RNF144**	204040_at	9781	2p25.2-p25.1	ring finger protein 144	0.3757	0.7833
**PTER**	218967_s_at	9317	10p12	phosphotriesterase related	0.3757	0.7891
**MPI**	202472_at	4351	15q22-qter	mannose phosphate isomerase	0.3746	0.7815
**DCN**	201893_x_at	1634	12q21.33	decorin	0.3724	0.7942
**ITGBL1**	205422_s_at	9358	13q33	integrin, beta-like 1 (with EGF-like repeat domains)	0.3712	0.7681
**SERPINF1**	202283_at	5176	17p13.1	serpin peptidase inhibitor, clade F (alpha-2 antiplasmin, pigment epithelium derived factor), member 1	0.3712	0.7836
**SH2D3A**	219513_s_at	10045	19p13.3	SH2 domain containing 3A	0.3679	0.8039
**VASH1**	203940_s_at	22846	14q24.3	vasohibin 1	0.3668	0.7925
**HLA-DPB1**	201137_s_at	3115	6p21.3	major histocompatibility complex, class II, DP beta 1	0.3645	0.8003

**ACADM**	202502_at	34	1p31	acyl-Coenzyme A dehydrogenase, C-4 to C-12 straight chain	0.3634	0.8029
**IGFBP4**	201508_at	3487	17q12-q21.1	insulin-like growth factor binding protein 4	0.3623	0.7776
**TNS3**	217853_at	64759	7p12.3	tensin 3	0.3623	0.7989
**RAI2**	219440_at	10742	Xp22	retinoic acid induced 2	0.3601	0.7602
**TRIM22**	213293_s_at	10346	11p15	tripartite motif-containing 22	0.3590	0.8100
**MGST2**	204168_at	4258	4q28.3	microsomal glutathione S-transferase 2	0.3579	0.8151
**AZGP1**	209309_at	563	7q22.1	alpha-2-glycoprotein 1, zinc-binding	0.3567	0.7795
**SCP2**	201339_s_at	6342	1p32	sterol carrier protein 2	0.3567	0.8178
**PLCD1**	205125_at	5333	3p22-p21.3	phospholipase C, delta 1	0.3567	0.8058
**DNAJC15**	218435_at	29103	13q14.1	DnaJ (Hsp40) homolog, subfamily C, member 15	0.3556	0.7838
**CFB**	202357_s_at	629	6p21.3	complement factor B	0.3556	0.7754
**FN1**	210495_x_at	2335	2q34	fibronectin 1	0.3545	0.8306
**ZNF33B**	215022_x_at	7582	10q11.2	zinc finger protein 33B	0.3534	0.8105
**RABGAP1L**	213982_s_at	9910	1q24	RAB GTPase activating protein 1-like	0.3534	0.7836
**HLA-DRA**	208894_at	3122	6p21.3	major histocompatibility complex, class II, DR alpha	0.3523	0.8058
**C1R**	212067_s_at	715	12p13	complement component 1, r subcomponent	0.3512	0.8093
**GNG12**	212294_at	55970	1p31.3	guanine nucleotide binding protein (G protein), gamma 12	0.3501	0.8122
**C1orf123**	203197_s_at	54987	1p32.3	chromosome 1 open reading frame 123	0.3501	0.8422
**SEPP1**	201427_s_at	6414	5q31	selenoprotein P, plasma, 1	0.3501	0.8133
**PARP3**	209940_at	10039	3p21.31-p21.1	poly (ADP-ribose) polymerase family, member 3	0.3501	0.8094
**SELENBP1**	214433_s_at	8991	1q21-q22	selenium binding protein 1	0.3489	0.8036
**PIGN**	219048_at	23556	18q21.33	phosphatidylinositol glycan anchor biosynthesis, class N	0.3445	0.7901
**NME5**	206197_at	8382	5q31	non-metastatic cells 5, protein expressed in (nucleoside-diphosphate kinase)	0.3445	0.7805
**COL1A2**	202403_s_at	1278	7q22.1	collagen, type I, alpha 2	0.3423	0.8226
**PPAP2C**	209529_at	8612	19p13	phosphatidic acid phosphatase type 2C	0.3423	0.8188

**PMP22**	210139_s_at	5376	17p12-p11.2	peripheral myelin protein 22	0.3423	0.8282
**MGP**	202291_s_at	4256	12p13.1-p12.3	matrix Gla protein	0.3400	0.8023
**COL3A1**	201852_x_at	1281	2q31	collagen, type III, alpha 1 (Ehlers-Danlos syndrome type IV, autosomal dominant)	0.3378	0.8234
**MICALL2**	219332_at	79778	7p22.3	MICAL-like 2	0.3378	0.8158
**APPL**	218158_s_at	26060	3p21.1-p14.3	adaptor protein containing pH domain, PTB domain and leucine zipper motif 1	0.3378	0.8363
**GATA3**	209602_s_at	2625	10p15	GATA binding protein 3	0.3367	0.7803
**COL10A1**	205941_s_at	1300	6q21-q22	collagen, type X, alpha 1(Schmid meta chondrodysplasia)	0.3367	0.7900
**LUM**	201744_s_at	4060	12q21.3-q22	lumican	0.3344	0.8116
**AGR2**	209173_at	10551	7p21.3	anterior gradient 2 homolog (Xenopus laevis)	0.3333	0.7684
**CDKN1B**	209112_at	1027	12p13.1-p12	cyclin-dependent kinase inhibitor 1B (p27, Kip1)	0.3333	0.8310
**CRIP1**	205081_at	1396	14q32.33	cysteine-rich protein 1 (intestinal)	0.3333	0.8013
**CTA-246H3.1**	209138_x_at	91353	22q11.23	similar to omega protein	0.3322	0.7845
**SOX9**	202935_s_at	6662	17q24.3-q25.1	SRY (sex determining region Y)-box 9 (campomelic dysplasia, autosomal sex-reversal)	0.3311	0.8100
**IL4R**	203233_at	3566	16p11.2-12.1	interleukin 4 receptor	0.3311	0.8272
**KRT19**	201650_at	3880	17q21.2	keratin 19	0.3300	0.8258
**CD74**	209619_at	972	5q32	CD74 molecule, major histocompatibility complex, class II invariant chain	0.3289	0.8277
**EFHD1**	209343_at	80303	2q37.1	EF-hand domain family, member D1	0.3255	0.8156
**RIN2**	209684_at	54453	20p11.22	Ras and Rab interactor 2	0.3255	0.8429
**CTDSP1**	217844_at	58190	2q35	CTD (carboxy-terminal domain, RNA polymerase II, polypeptide A) small phosphatase 1	0.3244	0.8356
**IGFBP7**	201162_at	3490	4q12	insulin-like growth factor binding protein 7	0.3233	0.8135
**ATP1B1**	201242_s_at	481	1q24	ATPase, Na+/K+ transporting, beta 1 polypeptide	0.3233	0.8208
**PSMB8**	209040_s_at	5696	6p21.3	proteasome (prosome, macropain) subunit, beta type, 8 (large multifunctional peptidase 7)	0.3233	0.8152
**ARHGDIB**	201288_at	397	12p12.3	Rho GDP dissociation inhibitor (GDI) beta	0.3222	0.8319

**CD24**	208650_s_at	934	6q21	CD24 molecule	0.3211	0.8320
**MMP2**	201069_at	4313	16q13-q21	matrix metallopeptidase 2 (gelatinase A, 72kDa gelatinase, 72kDa type IV collagenase)	0.3188	0.8152
**KRT18**	201596_x_at	3875	12q13	keratin 18	0.3188	0.8464
**KIAA0409**	203171_s_at	23378	11p15.4	KIAA0409	0.3166	0.8541
**POSTN**	210809_s_at	10631	13q13.3	periostin, osteoblast specific factor	0.3133	0.8452
**DENND2D**	221081_s_at	79961	1p13.3	DENN/MADD domain containing 2D	0.3133	0.8203
**ARID5B**	212614_at	84159	10q21.2	AT rich interactive domain 5B (MRF1-like)	0.3099	0.8451
**COL6A3**	201438_at	1293	2q37	collagen, type VI, alpha 3	0.3077	0.8301
**RIC8A**	221647_s_at	60626	11p15.5	resistance to inhibitors of cholinesterase 8 homolog A (C. elegans)	0.3066	0.8450
**DSP**	200606_at	1832	6p24	desmoplakin	0.3021	0.8475
**HLA-C**	208812_x_at	3107	6p21.3	major histocompatibility complex, class I, C	0.2988	0.8386
**STK16**	209622_at	8576	2q34-q37	serine/threonine kinase 16	0.2943	0.8430
**ZNF140**	204523_at	7699	12q24.32-q24.33	zinc finger protein 140	0.2943	0.8655

Table S2. Prognosis based average classification results of the IPRE algorithm in (a) 1,794 dataset, and (b) 2,268 dataset, with different cut-off points.

	Cut-off	TN	FP	TP	FN	SE (95% CI)	SP (95% CI)	ACC (95% CI)	F (95% CI)
**(a) 1,794 Dataset**	-0.6	897	0	214	683	0.239 (0.21 to 0.27)	1 (1 to 1)	0.619 (0.6 to 0.64)	0.385 (0.35 to 0.42)
	-1	892	5	343	554	0.382 (0.35 to 0.41)	0.994 (0.99 to 1)	0.688 (0.67 to 0.71)	0.551 (0.52 to 0.59)
	-1.2	878	19	456	441	0.508 (0.48 to 0.54)	0.979 (0.97 to 0.99)	0.744 (0.72 to 0.76)	0.665 (0.63 to 0.7)
	-1.4	834	63	530	367	0.591 (0.56 to 0.62)	0.930 (0.91 to 0.95)	0.760 (0.74 to 0.78)	0.711 (0.68 to 0.74)
	**-1.48**	**815**	**82**	**567**	**330**	**0.632 (0.6 to 0.66)**	**0.909 (0.89 to 0.93)**	**0.770 (0.75 to 0.79)**	**0.734 (0.7 to 0.76)**
	-1.6	764	133	573	324	0.639 (0.61 to 0.67)	0.852 (0.83 to 0.88)	0.745 (0.73 to 0.77)	0.715 (0.68 to 0.75)
	-1.8	672	225	616	281	0.687 (0.66 to 0.72)	0.749 (0.72 to 0.78)	0.718 (0.7 to 0.74)	0.709 (0.68 to 0.74)
	-2	569	328	653	244	0.728 (0.7 to 0.76)	0.634 (0.6 to 0.67)	0.681 (0.66 to 0.7)	0.695 (0.67 to 0.73)
	-2.2	453	444	709	188	0.790 (0.76 to 0.82)	0.505 (0.47 to 0.54)	0.648 (0.63 to 0.67)	0.692 (0.66 to 0.72)
	-2.7	3	894	824	73	0.919 (0.9 to 0.94)	0.003 (0 to 0.01)	0.461 (0.44 to 0.48)	0.630 (0.61 to 0.66)
**(b) 2,268 Dataset**	-0.6	1371	0	214	683	0.239 (0.21 to 0.27)	1 (1 to 1)	0.699 (0.68 to 0.72)	0.385 (0.35 to 0.42)
	-1	1283	88	323	574	0.360 (0.33 to 0.39)	0.936 (0.92 to 0.95)	0.708 (0.69 to 0.73)	0.494 (0.46 to 0.53)
	-1.2	1191	180	437	460	0.487 (0.45 to 0.52)	0.869 (0.85 to 0.89)	0.718 (0.7 to 0.74)	0.577 (0.54 to 0.61)
	-1.4	1044	327	501	396	0.559 (0.53 to 0.59)	0.761 (0.74 to 0.78)	0.681 (0.66 to 0.7)	0.581 (0.55 to 0.61)
	**-1.48**	**967**	**404**	**539**	**358**	**0.601 (0.57 to 0.63)**	**0.705 (0.68 to 0.73)**	**0.664 (0.65 to 0.68)**	**0.586 (0.55 to 0.62)**
	-1.6	864	507	577	320	0.643 (0.61 to 0.67)	0.630 (0.6 to 0.66)	0.635 (0.62 to 0.66)	0.583 (0.55 to 0.61)
	-1.8	733	638	616	281	0.687 (0.66 to 0.72)	0.535 (0.51 to 0.56)	0.595 (0.58 to 0.62)	0.573 (0.54 to 0.6)
	-2	607	764	660	237	0.736 (0.71 to 0.77)	0.443 (0.42 to 0.47)	0.559 (0.54 to 0.58)	0.569 (0.54 to 0.6)
	-2.2	491	880	709	188	0.790 (0.76 to 0.82)	0.358 (0.33 to 0.38)	0.529 (0.51 to 0.55)	0.570 (0.54 to 0.6)
	-2.7	7	1364	818	79	0.912 (0.89 to 0.93)	0.005 (0 to 0.01)	0.364 (0.34 to 0.38)	0.531 (0.51 to 0.56)

Here, TP defines true positive, TN defines true negative, FP defines false positive, FN defines false negative, ACC defines the accuracy of the algorithm, SE defines sensitivity, SP defines specificity, F defines the F-value, and 95% CI defines 95% confidence intervals. A cut-off

point of -1.480 (highlighted as bold) gives better results in terms of high SE, SP and ACC, compared with other cut-off points.

Table S3. Enriched biological process GO terms for the genes in our prognostic gene signature

GO	Description	*p*-value
GO:0050920	Regulation of chemotaxis	4.74E-05
GO:0002541	Activation of plasma proteins involved in acute inflammatory response	5.20E-05
GO:0050778	Positive regulation of immune response	6.71E-05
GO:0006958	Complement activation, classical pathway	3.54E-04
GO:0051605	Protein maturation by peptide bond cleavage	7.67E-04
GO:0006954	Inflammatory response	9.13E-04
GO:0009611	Response to wounding	9.19E-04
GO:0032103	Positive regulation of response to external stimulus	1.88E-06
GO:0043627	Response to estrogen stimulus	3.54E-04
GO:0050867	Positive regulation of cell activation	1.50E-04
GO:0008283	Cell proliferation	1.88E-06
GO:0009991	Response to extracellular stimulus	1.05E-04
GO:0010647	Positive regulation of cell communication	1.32E-04
GO:0042981	Regulation of apoptosis	2.08E-05
GO:0010941	Regulation of cell death	5.20E-05
GO:0006355	Regulation of transcription, DNA-dependent	1.50E-04
GO:0042325	Regulation of phosphorylation	1.05E-04

Table S4. Enriched pathway classes for the genes in our prognostic gene signature

Pathway description	*p*-value
Cell adhesion molecules (CAMs)	4.49E-04
Cell Cycle	5.74E-04
Integrin signalling pathway	2.08E-05
ECM-receptor interaction	1.88E-06
Axon guidance	5.74E-04
Signaling by PDGF	4.49E-04
Focal adhesion	1.88E-06
Signaling in immune system	3.53E-05
T cell activation	1.50E-04
Metabolism of lipids and lipoproteins	1.50E-04
ATM signaling	3.54E-04
Inflammation mediated by chemokine and cytokine signaling pathway	2.08E-05
TGF-beta signaling	5.20E-05
Pathways in cancer	4.49E-04
Apoptosis	1.08E-06

Supplementary Figures

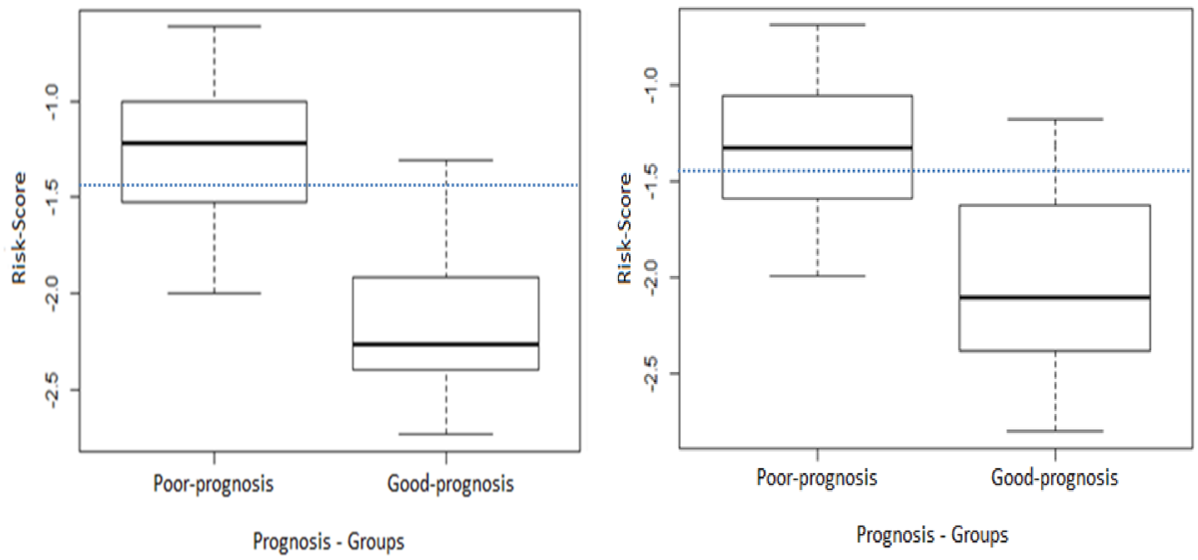

Figure S1. The boxplot of the IPRE algorithm for two prognosis groups, i.e., poor-prognosis and good-prognosis in the 1,794 dataset and the 2,268 dataset. The left figure represents the boxplot for the 1,794 dataset and the right figure represents the boxplot for the 2,268 dataset. Here, the x-axis represents the two prognosis groups, the y-axis represents the risk scores, and the dotted horizontal line represents our cut-off point i.e., -1.480. It can be clearly seen the two prognosis groups are separated in both datasets.

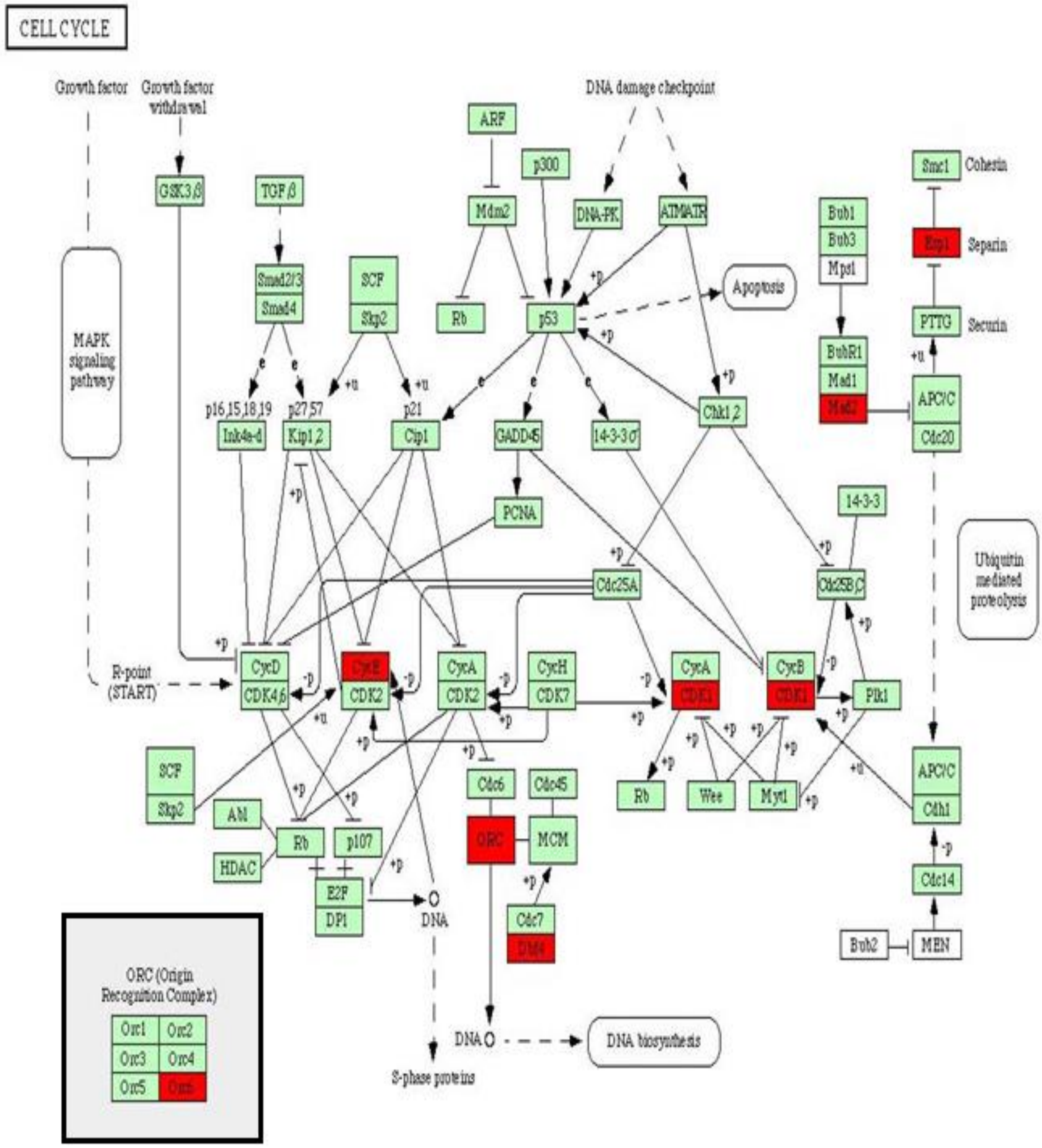

Figure S2. The KEGG pathway of a Cell Cycle. Here, the box highlighted red represents the genes in our gene signature. This figure was generated using the KEGG mapper [http://www.genome.jp/kegg/tool/map_pathway1.html].
